# Tranexamic acid in arthroscopic surgery: efficacy, safety, and clinical considerations

**DOI:** 10.3389/fsurg.2025.1679683

**Published:** 2025-11-19

**Authors:** Wei Wang, Qi ru Tian, Sheng Li

**Affiliations:** 1 Hainan Vocational University of Science and Technology, Haikou, Hainan, China; 2 Central Hospital Affiliated to Shenyang Medical College, Shenyang, Liaoning, China

**Keywords:** tranexamic acid, arthroscopic surgery, application, safety, blood loss

## Abstract

This study reviews the applications and effects of tranexamic acid in arthroscopic surgery. Bleeding during arthroscopic surgery is an important factor affecting surgical outcomes and postoperative recovery. Tranexamic acid is an anti fibrinolytic drug that can effectively inhibit fibrin degradation and may be helpful in reducing surgical bleeding and improving surgical field clarity. In this review, the pharmacological mechanism of tranexamic acid is first introduced, including its effect on the fibrinolytic system and its specific mechanism of action to reduce bleeding. Subsequently, the review describes the application of tranexamic acid in arthroscopic surgery, analyzes the safety of tranexamic acid in arthroscopic surgery, and related factors affecting the effectiveness of tranexamic in arthroscopic surgery. In addition, this review highlights future research directions regarding tranexamic acid in arthroscopic surgery. A comprehensive analysis of existing literature indicates that tranexamic acid has an impact on blood loss, pain, surgical time, surgical field clarity, and postoperative function during arthroscopic surgery. Therefore, the application of tranexamic acid in arthroscopic surgery has high clinical value and significance.

## 1 Introduction

**Figure 1:**
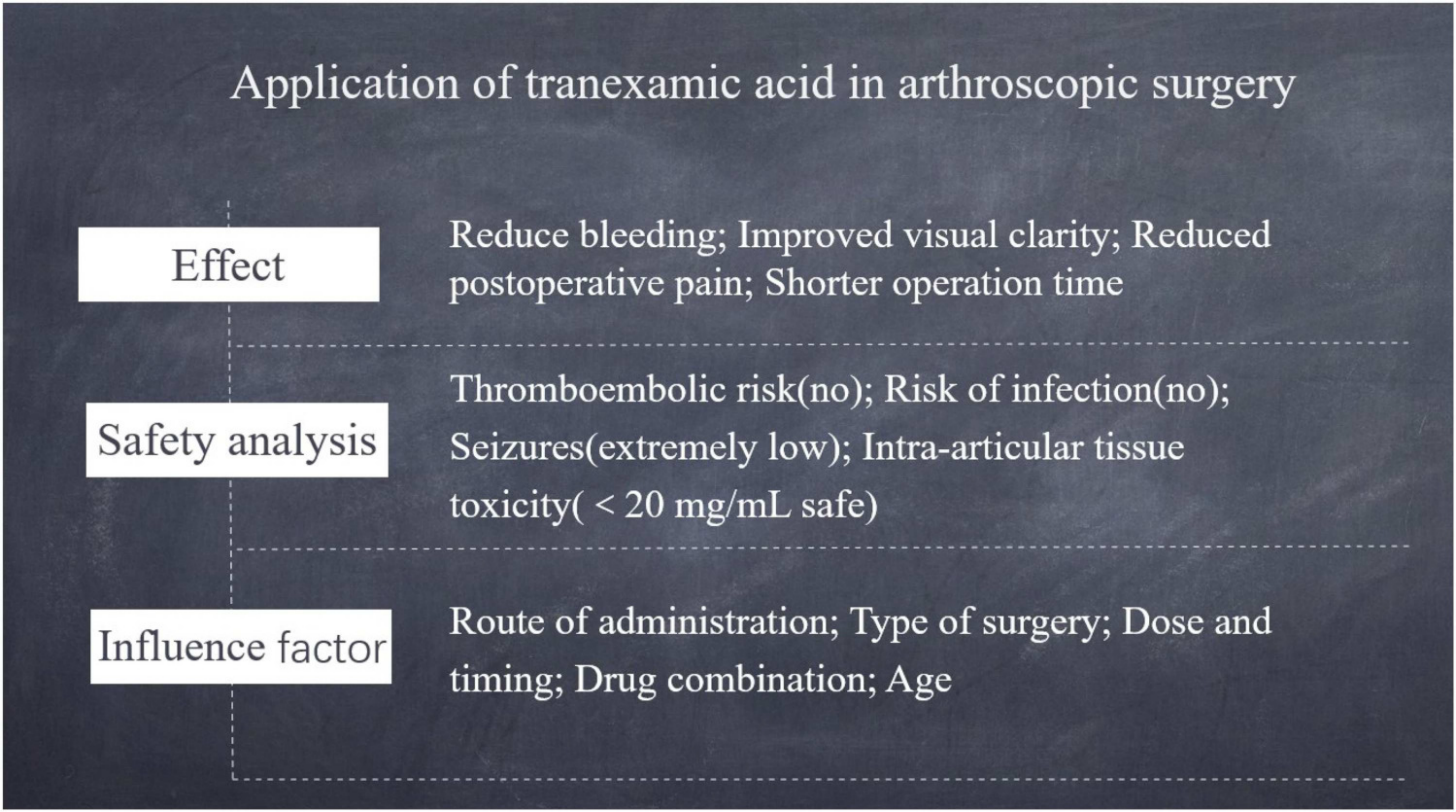
Summary of the application of tranexamic acid in arthroscopic surgery.

Arthroscopic surgery is a minimally invasive surgical approach (1) that occupies an important position in modern medicine. This surgical approach is not only minimally invasive and allows quick recovery (2), but also has a significant effect on the diagnosis and treatment of a variety of joint diseases. However, even in minimally invasive surgery, intraoperative and postoperative bleeding remain non-negligible problems (3), which may affect the clarity of the surgical field, increase the difficulty of surgery, and affect the speed of rehabilitation and surgical results of patients. Tranexamic acid, a synthetic amino acid antifibrinolytic agent, is widely used clinically to reduce or prevent bleeding caused by plasminogen activators. It enhances coagulation by inhibiting fibrin breakdown, thereby achieving hemostasis. In recent years, with the extensive study of tranexamic acid, its application in arthroscopic surgery has gradually received attention from the medical community (4). Although tranexamic acid has a significant effect on hemostasis, its specific application mode, dosage, and possible side effects in arthroscopic surgery need to be further explored. More clinical studies are required to provide evidence to support the balance between the hemostasis effects and potential risks. Therefore, the aim of this study was to systematically investigate the application effect and safety of tranexamic acid in arthroscopic surgery to provide a strong reference basis for clinical practice, further optimise surgery, and improve the quality of patient rehabilitation. Through an in-depth analysis of the mechanism of action of tranexamic acid, clinical applications, and possible risk factors, we expect to provide new ideas and methods for hemostasis strategies in arthroscopic surgery. The summary shown in Figure 1.

## 2 Methods

PUBMED was retrieved on October 16, 2025. The keywords are “tranexamic acid” and “arthroscopy”. A randomized controlled trial comparing TXA with placebo or other control drugs in patients undergoing arthroscopic shoulder, knee, and hip surgery over the past five years was included; And system overview. Select high-quality literature for review and summary, Table 1. Summary of Key Studies on Tranexamic Acid in Arthroscopic Surgery; Table 2. Safety analysis of tranexamic acid in arthroscopic surgery; And conduct a detailed analysis.

**Table 1:** Summary of Key studies on tranexamic acid in arthroscopic surgery.

Author, year, country	Level of evidence	Sample size	Surgery type	Dosage regimen	Blood loss (mL)	Pain	Operation time	Visual clarity	Function
Danielle Dagher, M.Sc.a, 2025, Canada	I	1,009	Shoulder Arthroscopy	Intravenous administration/irrigation solution/intra-articular injection	No difference	No difference (4.1)	No difference(91 min)	Slight increase (2.52)	–
Katherine A. Burns, MD, 2025, USA	Ⅱ	165	Shoulder Arthroscopy	Intravenous injection of TXA 1,000 mg	–	No differences were found	–	–	–
Jinkun Guo, 2025, China	Ⅱ	40	Shoulder Arthroscopy	Postoperative intra-articular injection of tranexamic acid (diluted to 20 mL with physiological saline)	–	No difference	–	–	Good early and mid-term functional recovery after surgery
Yushun Qian, 2024, China	I	1,068	Shoulder Arthroscopy	Seven studies involved intravenous route, three involved intra-articular injection, and one involved periarticular injection, with the most common dose being 1 gram.	No difference	No difference	There was no difference in overall surgical time, but subgroup analysis of randomized controlled trials showed a shortened surgical time for TXA.	Improve visual clarity	–
Neil Jain, 2024, USA	I	999	Shoulder Arthroscopy	Oral, intravenous or local use	–	No difference	–	Improve visual clarity	–
Mahdi Yousef Alyousef, 2024, Saudi Arabia	I	510	Shoulder Arthroscopy	1,000 mg IV 10 min preoperative	No difference	On the first day, the pain score significantly decreased	significantly reduce	No difference	–
Huihu Wang, 2024, China	Ⅱ	64	Shoulder Arthroscopy	Intravenous injection of 1,000 mg TXA (concentration: TXA 50 mg/mL) 10 min before surgery	Reduce blood loss	No difference	No difference	–	–
Hyeon Ju Shin, 2024, Republic of Korea	Ⅱ	63	Shoulder Arthroscopy	Inject a mixture of 100 mL TXA and physiological saline intravenously 10 min before surgery	–	–	–	Can improve visual clarity during intra-articular soft tissue surgery	–
Ryosuke Takahashi, 2023, Japan	Ⅱ	70	Shoulder Arthroscopy	Postoperative intra-articular injection of TXA (10 mL) and physiological saline (10 mL)	–	No difference	–	–	–
Rangteng Zhu, 2023, China	Ⅱ	162	Shoulder Arthroscopy	Postoperative intra-articular injection of 10 milliliters TXA (100 milligrams/milliliter)	Reduce total blood loss within 24 h after surgery	Reduce pain within 24 h after surgery	–	–	–
Shinji Kawaguchi, 2023, Japan	Ⅱ	129	Shoulder Arthroscopy	Preoperative TXA was administered intravenously at a dose of 1 g	Reduce hemoglobin loss on the 7th day after surgery	–	Reduce the total surgical time	Improve visual clarity	–
Mohammad Ayati Firoozabadi, 2025, Iran	Ⅱ	100	Knee arthroscopy	1: Randomly allocate a proportion of 1 to receive intra-articular injections containing TXA (1 g), morphine sulfate, ketorolac, lidocaine, and physiological saline + 15 mg/kg intravenous injection of TXA	–	Not significantly reducing early postoperative pain;	–	–	Improve functional recovery at 1 and 3 months after surgery
Junqiao Li, 2023, China	Ⅱ	87	Knee arthroscopy	Postoperative local administration of TXA (50 mL, 10 mg/mL)	Reduce postoperative blood loss	Reduce early postoperative pain	–	–	Improve early postoperative knee joint function
Weihao Sun, 2023, China	Ⅱ	80	Knee arthroscopy	Postoperative intra-articular injection of 50 mL TXA (1 g: 100 mL)	Reduce postoperative bleeding	Reduce early postoperative pain	No difference	–	No difference
Yuyan Na, 2022, China	I	418	Knee arthroscopy	Administer TXA at a recommended dose of 15 mg/kg 10 min before inflating the tourniquet, and continue intravenous infusion of 10 mg/kg/h for 3 h after surgery	TXA can reduce intra-articular bleeding	TXA can alleviate pain	–	–	–
Nedal Alkhatib, 2022, Canada	I	807	Knee arthroscopy	Compared with intra-articular TKA, intravenous injection of TXA can improve and prolong joint hemorrhage reduction	Reduce bleeding	Improve early pain	–	–	Improve early functionality
Tze Khiang Tan, 2021, Australia	I	580	Knee arthroscopy	Intravenous or intra-articular	Reduce bleeding	–	–	–	Improve joint range of motion
S. Andrew Samborski, 2025, USA	Ⅱ	862	Hip arthroscopy	Preoperative static TXA (single dose 1–2 g)	–	No difference	Less total surgical time	–	–
Kyle N. Kunze, M.D., 2025, USA	Ⅱ	78	Hip arthroscopy	Static point of 100 mL/0.9% physiological saline solution containing 1,000 mg TXA during surgery	–	–	–	No improvement in vision	–
Ning Li, 2021, China	Ⅱ	34	Hip arthroscopy	Preoperative intravenous infusion of 15 mg/kg TXA	Reduce blood loss	–	–	Improve surgical field of view	Promoting early and rapid recovery of hip joint function
Kyle Goldstein, 2022, Canada	I	724	Shoulder/Knee/Hip arthroscopy	Postoperative intra-articular injection; Preoperative intravenous (IV) infusion; Intraoperative intravenous injection	Reduce bleeding	Improve pain score 6 weeks after surgery	No loss of surgical time	Improve visual clarity	–

TXA, tranexamic acid; IV, intravenous.

A literature review on the administration regimen and related effects of tranexamic acid in shoulder, knee, and hip arthroscopic surgery in the past five years.

**Table 2:** Safety analysis of tranexamic acid in arthroscopic surgery.

Author, Year, Country	Complication			
	Thrombus	Infection	Epilepsy	Organizational toxicity
S. Andrew Samborski, 2025, USA	Will not increase risk	–	–	–
Neil Jain, 2024, USA	Will not increase risk	–	–	–
Huihu Wang, 2024, China	Will not increase risk	inhibit inflammation	–	–
Hyeon Ju Shin, 2024, Republic of Korea	Will not increase risk	–	–	–
Junqiao Li, 2023, China	Will not increase risk	Inhibit inflammatory markers; No infection	–	–
Weihao Sun, 2023, China	One case of deep vein thrombosis occurred	No infection	–	–
Yuyan Na, 2022, China	No venous thrombosis formation	No infection	–	–
Kyle Goldstein, 2022, Canada	No increase in venous thrombosis formation	No increase in infection	No	High dose TXA exhibits cartilage toxicity
Ning Li, 2021, China	No infection	No deep vein thrombosis formation	–	–
Hua Luo, 2023, China	–	–	Focal convulsion	–
Ming Wang, 2024, China	–	–	–	TXA concentration ≥ 50 mg/mL significantly reduces the viability of rat cartilage and meniscus cells
Daniel J Song, 2024, USA	–	–	–	High concentration TXA has cartilage toxicity
Hong Yu, 2024, China	Preventing venous thrombosis	–	–	TXA concentration of 15.6 mg/mL injected into the joint cavity has no significant effect on cartilage
Jiahao Wang, 2023, China	–	–	–	TXA exceeding 50 mg/mL can cause cartilage damage
Scott M Bolam, 2022, New Zealand	–	–	–	Local TXA treatment concentrations of 20 mg/mL or higher exhibit dose—and time-dependent cytotoxicity towards tendon derived cells and osteoblast like cells.

TXA, tranexamic acid.

## 3 Overview of tranexamic acid

### 3.1 Pharmacological properties and mechanism of action of tranexamic acid in brief

**Figure 2:**
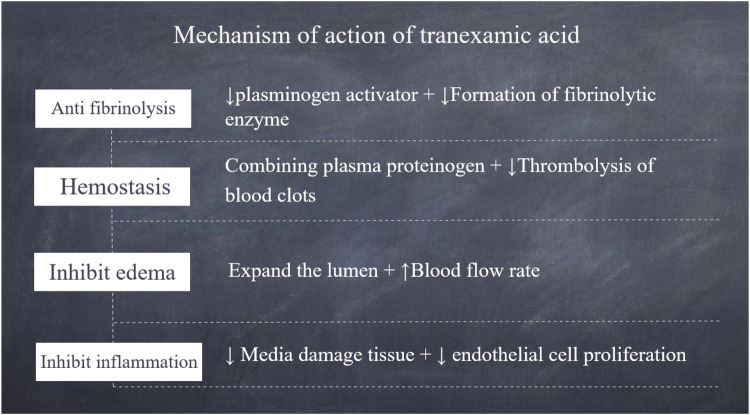
Schematic diagram of the mechanism of action of tranexamic acid.

Tranexamic acid is a synthetic lysine analogue, a white crystalline powder that is odourless and slightly bitter. It is freely soluble in water and exhibits good solubility in ethanol. It inhibits plasminogen activators (e.g., tissue-type plasminogen activator t-PA and urokinase-type plasminogen activator u-PA), reducing plasmin formation by competitive inhibition, thus exerting antifibrinolytic effects. It is a safe adjuvant drug that can be used in various gynaecological surgeries to reduce the risk of blood loss and need for blood transfusion (5), and is an effective and safe drug-based blood protection technique for the treatment of clinically significant bleeding (6) (Figure 2).

In addition to preventing fibrinolysis and blood loss, tranexamic acid has also been reported to inhibit inflammation and oedema after trauma. Tranexamic acid can act directly at gap junctions between endothelial cells and collagen bundles in the upper layer of the basement membrane. Fluid flows out of the gap and leads to luminal expansion, thereby accelerating blood flow velocity and playing a role in improving microcirculation. Tranexamic acid may also protect the endothelial and epithelial cell monolayers and stimulate mitochondrial respiration (7). Tranexamic acid can block the damage caused by various mediators produced during the inflammatory response to tissues, such as prostaglandin E, thereby reducing local oedema and the inflammatory response and promoting wound healing. Tranexamic acid inhibits and blocks the proliferation, migration, and angiogenesis of endothelial cells in the middle and late stages, and exerts anti-inflammatory and immunomodulatory effects through this pathway (7). In an animal experiment, it was mentioned that treatment of mice with tranexamic acid (16.1 ± 2.4/10^4^ μ m^2^) significantly inhibited the proliferation and migration of intravascular cells (8). TXA may help stabilize the intra-articular environment, exert anti-inflammatory and edema inhibiting properties by reducing fibrin deposition, limiting joint hemorrhage, and subsequently alleviating synovitis and joint stimulation (9). Research has shown that TXA can exert its anti-inflammatory effect by inhibiting the elevation of pro-inflammatory factors (TNF—α and IL-6) (10).

Tranexamic acid reduces plasmin and tyrosinase activities, thereby decreasing melanogenesis and skin pigmentation (11). Tranexamic acid is used to treat hyperpigmentation, and topical administration is the most favourable route. Topical administration of tranexamic acid is limited by the low permeability of the outer layer of the skin and low availability of target melanocytes. Tranexamic acid, which targets the epidermal layer, allows more drugs to act on melanocytes, which are its target sites. Novel drug delivery agents, such as liposomes, solid lipid nanoparticles, nanolipid carriers, and local beads, have the potential to achieve epidermal targeting. Epidermal targeting of tranexamic acid can help in superior delivery of the drug, making topical treatment more effective.

### 3.2 Review of the development of tranexamic acid and its other applications in the medical field

Tranexamic acid was invented by the female Japanese pharmacologist Okamoto in 1962; however, due to the social environment at that time (such as gender discrimination), it did not immediately receive due attention and clinical application. In 1968, a large international clinical study of tranexamic acid for the treatment of menorrhagia was conducted, marking the beginning of the real application of tranexamic acid in clinical practice. Since then, tranexamic acid has been widely used in clinical trials for patients with bleeding tendencies such as haemophilia, oral surgery, and orthopaedics. Because of its direct hemostasis effect and safety, tranexamic acid is included in the World Health Organization (WHO) Essential Drug List and has become a classic hemostasis drug for surgery.

Tranexamic acid is mainly used for the treatment of various haemorrhages caused by acute or chronic localised or systemic primary hyperfibrinolysis and is commonly used in traumatic or surgical haemorrhages of plasminogen activator organs, such as the prostate, urethra, lung, brain, uterus, adrenal gland, and thyroid gland. Tranexamic acid can also be used in other areas of the medical field; for example, it can be used to prevent or reduce bleeding after tooth extraction or oral surgery in haemophilia patients with factor VIII or factor IX deficiency and reduce complications due to bleeding from dental surgery (12).

## 4 Application of tranexamic acid in arthroscopic surgery

Arthroscopic surgery usually involves a narrow surgical field and intraoperative bleeding can seriously affect the surgical field and procedure (13). Therefore, reducing intraoperative bleeding is essential for improving the quality of the procedure. Several studies have shown that tranexamic acid effectively reduces intraoperative bleeding and drainage during arthroscopic surgery. A meta-analysis showed that the use of tranexamic acid can significantly reduce the total blood loss and postoperative drainage volume of arthroscopic surgery compared to the control group (14), Tranexamic acid can be used in shoulder surgery to reduce perioperative blood loss, and the use of tranexamic acid may have other beneficial effects, including reduced postoperative pain and shorter operation time. Another interim analysis also found that tranexamic acid significantly improved the operative field and shortened the operative time for arthroscopic rotator cuff repair without increasing the incidence of adverse events (15). A systematic review and meta-analysis of randomised controlled trials found that the use of tranexamic acid significantly improved pain scores 6 weeks after surgery, reduced drainage volume, reduced the need for arthrocentesis, reduced the incidence of hemarthrosis, improved visual clarity and technical simplicity, and did not increase the incidence of other complications or extend surgery time (16). These findings suggest that tranexamic acid may be a useful adjunct to arthroscopic knee and shoulder surgery. Studies have shown that topical intra-articular application of tranexamic acid is effective in reducing postoperative bleeding and early postoperative pain in patients undergoing arthroscopic synovectomy for pigmented villonodular synovitis of the knee (17). The use of intravenous tranexamic acid in anterior cruciate ligament reconstruction surgery can reduce joint drainage and hemarthrosis and improve pain scores and range of motion in the early postoperative period, facilitating rehabilitation without increasing complications or thromboembolic events (18). However, studies on the efficacy of tranexamic acid in arthroscopic surgery are limited. A meta-analysis of shoulder arthroscopy found that tranexamic acid did not increase complications or thromboembolic events, but did not significantly improve visual field definition, pain scores, procedure time, or amount of irrigation (19). The results of a meta-analysis suggest that intravenous tranexamic acid reduces drainage volume, the need for knee aspiration, and knee swelling. In addition, it has positive short-term effects on clinical and functional outcomes. However, the high risk of bias, low study quality, and heterogeneity greatly reduced the quality of evidence and validity of the study conclusions. The authors concluded that the routine use of tranexamic acid in arthroscopic surgery is not recommended based on the current evidence (4).

We searched the database and summarized high-quality literature from the past five years, including the administration regimen of tranexamic acid in shoulder arthroscopy, knee arthroscopy, and hip arthroscopy surgery, blood loss, pain, surgery time, visual field clarity, and postoperative functional recovery. Please refer to Table 1 for details. The recommendations are as follows:

Shoulder arthroscopy group: The commonly used medication regimen is intravenous injection of 1,000 milligrams 10 min before surgery or/and intra-articular injection of TXA (10 mL) and physiological saline (10 mL) after surgery; May reduce the total blood loss in the early postoperative period; Basically unable to relieve pain; There was no significant difference in overall surgical time; Improve visual clarity; There is basically no impact on the postoperative recovery of shoulder joint function.

Knee arthroscopy group: The commonly used medication regimen is postoperative local administration of TXA (50 mL, 10 mg/mL); Reduce postoperative blood loss; Improve early pain; No impact on the surgical time; Has no impact on visual clarity; Improve early postoperative knee joint function.

Hip arthroscopy group: The commonly used medication regimen is 100 mL/0.9% saline containing 1000 mg TXA intravenously during surgery and/or preoperative intravenous injection of 15 mg/kg TXA; May be helpful in reducing bleeding; May be helpful in relieving pain; No reduction in surgical time; Improve visual clarity; May promote early and rapid recovery of hip joint function.

## 5 Safety analysis of tranexamic acid use in arthroscopic surgery

### 5.1 Thromboembolic risk

Several studies have shown that there is no increased risk of thromboembolic events, such as deep vein thrombosis or pulmonary embolism, in the tranexamic acid group compared with the control group. Patients undergoing arthroscopy, particularly arthroscopic meniscectomy, arthroscopic anterior cruciate ligament reconstruction, and arthroscopic rotator cuff repair, had improved outcomes and reduced complications related to thrombosis in the early postoperative period compared with non-tranexamic acid patients (20). A meta-analysis also found no significant difference in the incidence of deep vein thrombosis between the two groups (21). Thus, the available evidence supports the use of tranexamic acid in arthroscopic procedures without increasing the thromboembolic risk. See Table 2 for details.

### 5.2 Risk of infection

Several studies have reported no increased risk of postoperative infection after tranexamic acid administration. A retrospective cohort study (22) assessed the effects of tranexamic acid on the risk of infection after primary shoulder arthroplasty. The study, which included 9,276 patients, compared the risk of requiring revision surgery for deep infection within 5 years between patients who received preoperative intravenous tranexamic acid and those who did not, and showed no significant difference between the two groups (hazard ratio 1.00, 95% CI: 0.56–1.80). In addition, a cost-effectiveness analysis study (23) assessed the economic plausibility of using tranexamic acid to prevent joint infections in total shoulder arthroplasty. The results showed that if tranexamic acid reduced the infection rate by 0.009%, its use would be cost-effective. This study provides some theoretical support for tranexamic acid in preventing infection during joint surgery; however, direct clinical evidence is lacking. See Table 2 for details.

### 5.3 Seizures

A study reported a rare case of focal convulsions in a middle-aged male after topical application of tranexamic acid and intravenous infusion during spinal surgery (24). This article reviews relevant cases of adverse reactions to topical tranexamic acid during spinal surgery and discusses the possible mechanisms of TA-induced convulsions. The mechanism of TXA induced epilepsy and muscle spasms is mainly through TXA's direct inhibition of gamma aminobutyric acid and glycine receptors located at the postsynaptic site of spinal dorsal horn neurons, thereby increasing excitability. Therefore, it is advisable not to inject TXA locally into cerebrospinal fluid during intervertebral foramen endoscopy to avoid complications of epilepsy. And emphasize that this is a rare adverse event with low risk at typical arthroscopic doses, as shown in Table 2.

### 5.4 Intra-articular tissue toxicity

A review found that tranexamic acid was cytotoxic to chondrocytes, tenocytes, synoviocytes, and cells of periosteal origin at concentrations exceeding 20 mg/mL (25). Two-dimensional cell culture are more susceptible to tranexamic acid than three-dimensional and tissue block cultures. However, no significant toxic effects were found in *in vivo* studies, possibly due to concentration differences. The *in vitro* cytotoxic concentration (20 mg/mL) is significantly higher than the typical intra-articular concentration achieved in clinical practice (typically 10–50 mg in 20–30 mL of physiological saline, resulting in a concentration of approximately 0.3–2.5 mg/mL). Research has shown that intra-articular injection of 15.6 mg/mL TXA concentration has no significant effect on cartilage At 3, 6, and 12 months postoperatively, magnetic resonance imaging showed no statistically significant difference in cartilage signal values between the experimental group and the control group (26); TXA induces apoptosis in chondrocytes by activating endoplasmic reticulum stress, particularly in the 50 mg/mL and 100 mg/mL groups (27); Local TXA therapy has shown dose—and time-dependent cytotoxicity against tendon derived cells and osteoblast like cells at concentrations of 20 mg/mL and above in isolated 2D and 3D *in vitro* cultures. Based on these findings, concentrations below 20 mg/mL are expected to be safe, and orthopedic surgeons should be cautious when considering local TXA therapy, especially in soft tissue and non cemented joint replacement surgeries (28). Therefore, concentrations of 20 mg/mL or less are expected to be safe, but further clinical studies are required to assess their long-term safety. See Table 2 for details.

## 6 Factors affecting the effect of arthroscopic surgery using tranexamic acid

### 6.1 Importance of route of administration on the effect of tranexamic acid in arthroscopic surgery

Several studies (15, 16, 29, 30) have demonstrated that intravenous tranexamic acid can significantly improve surgical field definition, reduce blood loss and postoperative pain. Nevertheless, the effect of local intra-articular injection of tranexamic acid is controversial, and an *in vitro* study (31) suggested that high concentrations of tranexamic acid may be cytotoxic to chondrocytes. Therefore, current evidence supports intravenous administration as a safe and effective route of administration. A prospective observational study (32) found that the combination of intravenous and intra-articular administration of tranexamic acid further reduced intraoperative blood loss and shortened hospital stay compared with a single route of administration without increasing the risk of complications. Another randomised controlled trial (30) also found that this combination improved the surgical field better than a single route of administration. Therefore, this combination (IV + intra-articular) may be the optimal mode of administration, optimal dosing is not yet standardized. The specific dosage can refer to Table 1.

### 6.2 Type of surgery may affect the efficacy of tranexamic acid

A systematic review (19) found that the use of tranexamic acid in shoulder rotator cuff repair failed to improve the operative field or reduce pain. In knee arthroscopy (20, 31) and other arthroscopic procedures (16), tranexamic acid significantly improved the postoperative visual field, pain, and swelling. This may be related to the amount of bleeding and the method by which the procedure is performed.

### 6.3 Dose and timing of tranexamic acid administration

Although most studies have used intravenous administration, the optimal dose of tranexamic acid, and timing of administration (preoperative, intraoperative, or postoperative) need to be further explored (15). Different types of surgeries may require different dosing regimens to achieve optimal efficacy.

### 6.4 Combination of tranexamic acid with other hemostasis measures (e.g., epinephrine)

Some studies have investigated the effects of tranexamic acid in combination with other hemostasis agents, such as epinephrine. Intravenous tranexamic acid is not an effective alternative to epinephrine for irrigating fluid to improve vision in routine shoulder arthroscopy and is not used additively (33). However, whether there is a synergy between these measures and how to optimise the combination regimen requires further study.

### 6.5 Age

A secondary analysis of the CRASH-2 trial (34) found that trauma-related mortality increased with age; however, the treatment effect of tranexamic acid administration in different age groups did not appear heterogeneous.

## 7 Future research directions

7.1 A large-scale, multicentre, randomised controlled trial to assess the effect of tranexamic acid at different doses, routes, and timings in different types of arthroscopic surgery, and to investigate its impact on long-term prognosis and complications.

7.2 Studies using tranexamic acid in combination with other hemostasis measures (e.g., epinephrine) to assess the synergy between the two and to optimise the combination regimen.

7.3 Basic studies to investigate the effects of tranexamic acid on articular chondrocytes and synovial fluid to assess the safety of intra-articular administration.

7.4 Inclusion of baseline characteristics of the patients (such as age and body mass index, etc.) in the analysis to investigate the different effects of tranexamic acid in different populations.

7.5 A cost-effectiveness analysis to assess the economic value of tranexamic acid in arthroscopic surgery.

## 8 Conclusions

Tranexamic acid is a safe and effective anti fibrinolytic agent, and its effects on arthroscopic surgery may include reducing bleeding, relieving pain, affecting surgical time, improving visual field, and enhancing postoperative function. Intravenous administration is a safer and more effective route of administration, and combination therapy may provide the best results. The benefits of TXA are specific to surgery, and the heterogeneity of evidence rules out general recommendations. TXA is a promising adjunct, that its use should be individualized and cautious, and that its routine adoption awaits confirmation from large-scale, high-quality RCTs focusing on long-term efficacy and safety. Evidence heterogeneity and small sample sizes preclude routine TXA use across all arthroscopic procedures.Limitations of Current Evidence.

This article tends to be a narrative review, with a low level of evidence and a lack of transparency and reproducibility.

This manuscript mainly emphasizes the positive effects of tranexamic acid (TXA) in arthroscopic surgery, while downplaying or briefly mentioning conflicting findings.

This review did not fully address the high heterogeneity of the included studies in terms of patient demographics, surgical types, TXA doses, timing, and administration routes. This limits the universality of the conclusion, and its limitations should be clearly discussed.

Although short-term benefits and safety have been discussed, the long-term effects of TXA, particularly on cartilage health, joint function, and delayed complications, have not been fully addressed. Given that *in vitro* evidence suggests the possibility of cartilage toxicity at higher concentrations, this is a significant gap.
